# Depression and Metabolic Syndrome: A Narrative Review

**DOI:** 10.7759/cureus.22153

**Published:** 2022-02-12

**Authors:** Yousef Al-Khatib, Muhammad Adeel Akhtar, M. Ali Kanawati, Rumbidzai Mucheke, Maria Mahfouz, Maysan Al-Nufoury

**Affiliations:** 1 Orthopaedics, Royal College of Surgeons in Ireland, Dublin, IRL; 2 Trauma and Orthopaedics, Royal College of Surgeons in Ireland, Dublin, IRL; 3 Internal Medicine, Royal College of Surgeons in Ireland, Dublin, IRL; 4 Operating Department Practice, University of Huddersfield, Huddersfield, GBR; 5 Medicine, Royal College of Surgeons in Ireland, Dublin, IRL; 6 Psychiatry, The Spinney Hospital, Manchester, GBR

**Keywords:** depression and diabetes, cardiovascular complications, metabolic syndrome, depression, obesity

## Abstract

We reviewed the literature to investigate the relationship between depression and metabolic syndrome. Major depressive disorder is characterized by a low mood or a loss of interest for longer than two weeks. Metabolic syndrome describes multiple metabolic risk factors including obesity, insulin resistance, dyslipidemia, and hypertension. We divided our findings into environmental, genetic, epigenetic, and biological pathway links between depression and the different aspects of metabolic syndrome. We found various sources linking obesity and metabolic syndrome genetically, environmentally, biological pathway-wise, and, while not fully explored, epigenetically. Diabetes and depression were also found to be linked environmentally with both conditions increasing the risk of the other. Depression was also shown to be linked to cardiovascular complications as it increased the risk of occurrence of such complications in healthy people. These findings have led us to believe that there is a link between depression and metabolic syndrome on various levels, especially obesity.

## Introduction and background

Depression is a very common and complex disorder, which is more prevalent in women, and its causes are multifaceted [1]. Major depressive disorder is categorized as a depressed mood or a loss of interest or pleasure in daily activities for more than two weeks. Additionally, five of the nine specific symptoms must be present nearly every day, which include depressed mood or irritability, decreased interest in most activities, significant change in weight and appetite, change in sleep, psychomotor agitation or retardation, fatigue, feelings of worthlessness, diminished concentration, and suicidal thoughts [2]. The course of depression is recurrent as patients go through periods of symptomatic episodes and periods of recovery [1]. As reported by the World Health Organization, an estimated 350 million people of all ages suffer from depression globally. It is the leading cause of disability and is a major contributor to the overall global burden of disease [3]. In the USA, for the year 2000, the economic burden of depression was estimated at $83.1 billion, whereas in Europe, the annual cost of depression was €118 billion for the year 2004 [4]. It has also been consistently associated with metabolic syndrome, which is an overarching term for obesity, cardiovascular complications, and diabetes [5].

In the last decade, a lot of research has been done to elucidate the relationship between depression and metabolic syndrome, and most of the studies have found a positive association [6-16]. Although Foley et al. found no significant differences in metabolic outcomes of individuals with or without a history of major depression and also found no effect of gender on risk for metabolic syndrome [17], a study looking at literature from 2000 to 2011 regarding the prevalence of metabolic syndrome in patients with depressive disorder found that the prevalence of metabolic syndrome in patients with depression is high, although the prevalence rate varied among the analyzed studies [18]. In 2012, a meta-analysis of cross-sectional and prospective cohort studies evaluating the association between depression and metabolic syndrome also found that a bidirectional association between depression and metabolic syndrome exists [19]. More recently, Vancampfort et al. conducted a meta-analysis to clarify the prevalence and correlates of metabolic syndrome in people with major depressive disorder. In their sample size of 5,531 depressed individuals, 30.5% of them met the criteria for metabolic syndrome. Furthermore, when these individuals were compared with age- and gender-matched control groups, individuals with the major depressive disorder had a higher metabolic syndrome prevalence with an odds ratio of 1.54 [20]. These findings were replicated in another meta-analysis done in 2015 by the same group in which they found that the pooled metabolic syndrome prevalence in people with severe mental illness was 32.6% (95% CI: 30.8-34.3%; N = 198; n = 52,678) [21]. Furthermore, using the 2009-2010 National Health and Nutrition Examination Survey, Rethorst et al. showed that over 29% of depressed individuals had elevated levels of C-reactive protein and 41% met the criteria for metabolic syndrome. Most importantly, their data indicated that individuals with elevated inflammation are more likely to be obese and meet the criteria for metabolic syndrome [22] Other recent studies have also found a positive association between depression and metabolic syndrome [23-25].

This narrative review aims to present a comprehensive review of the literature regarding the relationship between depression and metabolic syndrome, with a specific focus on the shared epidemiology, environmental factors, biological pathways, and genetic factors of depression and obesity.

Methods

An electronic database search of PubMed was performed on August 21, 2021. The search terms included variations of the following keywords with the use of different Boolean operators: obese, obesity, morbid obesity, leptin, alpha-ketoglutarate-dependent dioxygenase FTO, adiposity, hyperphagia, high body-mass index, high BMI, weight gain, weight loss, weight control, overweight, body weight, waist-hip ratio, skinfold thickness, body adiposity index, weight cycling, overeat*, adipos*, depression, depressive disorder, major depressive disorder, adjustment disorders, depress*, psychological distress, low mood, unhappiness, melancholy, adult, and young adult.

Articles not published in English were excluded. The search returned 5,142 articles after which a title and abstract screening was done by two independent reviewers. The aim was to exclude any articles that did not discuss the relationship between obesity and depression along with their comorbidities of metabolic syndrome. All disagreements between the independent reviewers were resolved through discussion with a third reviewer and consensus opinion. This resulted in the exclusion of 4,139 articles and the final number of retained articles was 1,003 with a kappa of 0.56.

## Review

Depression and obesity

In 1976, Crisp et al. found a significant positive association between substantial obesity and low levels of depression in men from the suburbs and replicated the same findings in 1980 in a rural population [26,27]. However, the majority of studies done after that failed to replicate the findings [28-33]. Roberts et al. investigated the association between obesity and depression using data from a community-based study (Alameda County Study, 1994-1995). Although their results did not indicate obesity as a risk factor for depression, their results did suggest an association between obesity and depression [34]. More importantly, they found no evidence for the jolly fat hypothesis in their results (obesity reduces the risk of depression). Similarly, Dixon et al. examined depression before and after surgically induced weight loss. Using Beck Depression Inventory (BDI) questionnaires to measure depression, they found that high BDI scores correlated with poorer physical and mental quality-of-life measures. Their results also indicated that weight loss was associated with a significant and sustained fall in BDI score [35]. Another study in 2007 looked to examine self-reported physical disorders in people with recurrent depression compared with a psychiatrically healthy control group. Using 1,546 participants with recurrent depression and 884 controls, they found that obesity was more prevalent in the cases group than the control group [36]. Likewise, a study using a much larger sample size of 41,654 participants from the National Epidemiologic Survey on Alcohol and Related Conditions confirmed an association between obesity and depression, as well as several other psychiatric disorders [37]. A more recent study examining the relationship between elevated depression symptoms or stress and weight loss found that individuals with metabolic syndrome or elevated depression symptoms are less likely to lose significant weight [38].

Several studies have investigated if the relationship between depression and obesity is reciprocal. Roberts et al. examined the temporal relation between obesity and depression to determine if each constitutes a risk factor for the other. Using a two-wave (1994, 1999), five-year observational study design, they found that obesity at baseline was associated with increased risk of depression five years later; however, depression did not increase the risk of future obesity [39]. Similarly, Faith et al. reviewed population-based studies and found a significant amount of evidence to prove that obesity is prospectively associated with increased depression, with less consistent evidence that depression leads to obesity [40]. However, in 2010, de Wit et al. conducted a meta-analysis on community-based studies to examine the nature of the association between depression and obesity. They found that individuals with obesity (BMI > 30 kg/m2) were 1.18 times more likely to have depressive symptoms than individuals without obesity. After conducting a subgroup analysis, they found that this association was more clearly present in females than in males [41]. The same group conducted a meta-analysis of longitudinal studies to determine the relationship between depression, overweight, and obesity. Their findings confirmed a reciprocal link between depression and obesity, and they found that the association was more pronounced in Americans than Europeans. This could possibly be due to different sociocultural mechanisms across cultures and a higher mean adult BMI in the United States [42]. Additionally, a more recent study looking to review the relationship between obesity and mood disorders found that the relationship is bidirectional and convergent. This may be due to the shared risk factors such as chronic psychosocial stress, inadequate diet, lack of exercise, adverse socio-economic situations, and childhood trauma [43].

Moreover, some studies have shown that depression or obesity during childhood may increase the risk of becoming obese or depressed in adulthood, respectively. Hasler et al. found that among women, depressive symptoms before 17 years of age were associated with increased weight gain (4.8 vs. 2.6% BMI increase per 10 years), representing a greater risk for adult obesity (hazard ratio = 11.52; P < 0.05). Among men, only after controlling for confounders, depressive symptoms before 17 years of age were associated with increased weight gain (6.6 vs. 5.2% BMI increase per 10 years) in adulthood but not with the occurrence of obesity [44]. Similarly, Herva et al. examined the association between body size and depression in young adults at the age of 31 years. They concluded that obesity in adolescence may be associated with later depression in young adulthood and being overweight or obese both in adolescence and adulthood may be a risk for depression among female subjects [45].

Furthermore, Heo et al. investigated whether the association between depressive mood and obesity differs as a function of sex, age, and race in US adults after controlling for socio-economic variables of marital status, employment status, income level, and education level. They found that the relationship between depressive mood and obesity is dependent upon gender, age, and race. Young obese women, Hispanics in particular, are much more prone to depressive moods than non-obese women [46]. Another study examined the relationship between measures of adiposity and depressive symptoms in a large community-based sample. They found that visceral adipose tissue was significantly associated with an increase in depression amongst women; however, this association was not significant amongst men [47]. Likewise, another study done on adults in Puerto Rico in 2013 showed that depressive symptoms are associated with obesity, and this association is more significant in women, especially those with less education, severe obesity, and low income [48]. More recently, Molyneaux et al. conducted a meta-analysis to evaluate the prevalence and risk of antenatal and postpartum mental disorders among obese and overweight women. They found that obese pregnant women are at higher risk to experience elevated antenatal and postpartum depressive symptoms than normal-weight women, with an intermediate risk for overweight women [49].

Interestingly, some studies have shown that atypical depression (increased appetite and weight gain) is more likely to be associated with obesity than other types of depression. A study in 2012 found that subjects with atypical depression had significantly higher obesity rates compared to controls and individuals with classic depression (decreased appetite and weight loss). However, they found no significant difference in obesity among individuals with classic depression and non-depressed controls [50]. Similarly, another study investigated the rate of obesity associated with different types of depression amongst older adults (60 years or above) in the United States. After adjusting for sex, age, marital status, race, and personal income, the rate of obesity was significantly higher among individuals with atypical depression than those with classic, undifferentiated depression, or without depression [51]. Furthermore, McElroy et al. did a literature review for the years 1966-2003 to better inform mental health professionals about the overlap between mood disorders and obesity. They found that the most rigorous clinical studies suggest that children and adolescents with major depressive disorder may be at increased risk of becoming overweight. Additionally, the community studies suggested that depression with atypical symptoms in females is significantly more likely to be associated with overweight than depression with typical symptoms [52].

Additionally, several studies have found depression and obesity to follow a U-shaped pattern. A study in 2009 looked to examine the association between depression and BMI using data collected from 177,047 participants in the 2006 Behavioral Risk Factor Surveillance System. After adjusting for demographics, obesity-related comorbidities, lifestyle, or psychosocial factors, they found that men who had a BMI > 40 kg/m2 or BMI < 18.5 kg/m2 were significantly more likely to have current depression or lifetime diagnosed depression compared to men with normal BMI. Moreover, they found that women who were either overweight or obese were significantly more likely to experience depression than women with normal BMI [53]. Similarly, another study looked to explore the prevalence of depression among underweight, normal weight, overweight, and obese general practice patients. By conducting a cross-sectional survey in 12 Australian general practices, they found that weight and depression demonstrated a U-shaped relationship, with a higher prevalence of depression among underweight and obese individuals [54]. This pattern was replicated in another study that examined the comorbidity and direction of association between BMI and depressive symptoms. They found that obese and underweight individuals were at 1.3-2.1 and 1.5-2.3, respectively, times the risk of depression compared with normal weight [55]. Likewise, Noh et al. also found a U-shaped relationship between BMI and depression as the highest level of depressive symptoms was more prevalent in underweight and severely obese individuals [56].

Depression and diabetes along with cardiovascular complications

Although most of the studies have been done to elucidate the relationship between depression and obesity, some studies have also looked into the relationship between depression and diabetes along with cardiovascular complications.

A recent study evaluated the interaction between depressive symptoms and risk for type 2 diabetes. They found that participants with both depressive symptoms and metabolic dysregulation had the highest risk of diabetes (adjusted odds ratio = 6.61; 95% CI: 4.86-9.01), compared with those without depressive symptoms and metabolic dysregulation. Furthermore, the risk of diabetes in individuals with depressive symptoms and without metabolic dysregulation did not differ from the reference group. Whereas the adjusted odds ratio was 4.40 for those who had metabolic dysregulations and no depressive symptoms. These findings highlight the interaction between depressive symptoms and metabolic dysregulation as a risk factor for type 2 diabetes [57]. Similar findings were seen in another study in which participants with type 2 diabetes were at more risk for having a lifetime of major depression [58]. However, another study failed to find any significant association between depressive symptoms and diabetes among Chinese women; however, this may have been due to the study design and the imbalance in the number of participants in each group [59].

Samaan and MacQueen found that depression increases the risk of cardiovascular disease by 1.5-2 times in healthy individuals. Additionally, depression in cardiovascular disease patients increases the risk of cardiac morbidity and mortality by 1.5-2.5 times. Furthermore, migraine worsens the impacts on patients’ health and quality of life compared with the presence of depression alone [60].

Environmental factors

Depression and Obesity

While investigating the contribution of environmental factors in the co-occurrence of obesity and depression, Choy et al. found a consistent and significant positive environmental correlation between the lean mass index and symptoms of depression in men, suggesting that both lean mass index and symptoms of depression in men are affected through the same environmental factors. They found physical activity to be an evident candidate as it increases lean mass index by acquiring more muscles and decreases symptoms of depression by maintaining a low base of endorphin levels [61]. Another study found that adverse childhood experiences may be a shared environment for both obesity and depression [62]. Additionally, eating, physical activity, teasing, disordered eating, and stress may act as mediating factors for both conditions [63]. A systematic review done by Preiss et al. also found educational attainment, body image, binge eating, physical health, psychological characteristics, and interpersonal effectiveness to be consistently associated with obesity and depression [64]. Furthermore, many obese individuals experience low self-esteem and diminished self-efficacy, which have repeatedly demonstrated reliable correlations with negative affect, depressed mood, and clinical depression [65].

Depression and Type 2 Diabetes

Chronic stress was also found to be a common environmental factor between depression and type 2 diabetes as it can alter physiological systems such as inflammation and glucose metabolism [66]. Another study found strong evidence that intrauterine environment and birth weight can predispose individuals to type 2 diabetes. Additionally, childhood adversity, neighborhood environment, and poverty influence the predisposition to depression and diabetes as poor physical and social environments along with social capital are associated with worse diet and lower physical activity patterns [67]. Furthermore, in a meta-analysis done by Vancampfort et al., they found that higher levels of sedentary behavior are associated with a 112% increase in the relative risk of type 2 diabetes and a 147% increase in the relative risk of cardiovascular disease, a 90% increase in the risk of cardiovascular mortality, and a 49% increase in the risk of all-cause mortality [68]. Additionally, alcohol consumption, social support, access to health care, and socioeconomic status can also be shared modulators of depression, obesity, and diabetes [69].

Depression and Cardiovascular Complications

In a study done by Rottenberg et al., it was seen that kids who were depressed and had cardiovascular diseases were more overweight, had lower levels of regular physical activity, higher levels of sedentary lifestyles compared to controls, and were regular smokers [70].

Biological pathway

Metabolic Disorders

In recent years, several genome-wide investigations of obesity and associated disorders have revealed novel biological pathways that propagate the condition. Genome-wide expression profiling of obese individuals, compared to matched controls, revealed inconsistencies in several blood transcriptomes [71]. In particular, gene-set enrichment pathway analysis demonstrated increased transcription of genes in “ribosome,” “apoptosis,” and “oxidative phosphorylation pathways” in obese patients. These findings are consistent with the expected increase in energy demands, as well as cell death due to lipotoxic stimuli, due to obesity [71].

In another recent study, data from 1,000 unrelated genome-wide association studies on obesity were meta-analyzed in a biological-pathway analysis using gene-set enrichment algorithms. The vasoactive intestinal peptide was amongst those most significantly associated with obesity (nominal P = 0.0009) and BMI (nominal P = 0.0006).

Depression

The pathophysiological pathways of depression are expectedly complex and unclear, primarily due to its clinical and etiological heterogeneity [72].

Several studies have investigated the role of the hypothalamic-pituitary-adrenal (HPA) axis in the neuropathology of depression [72-75]. Dysregulation of this axis, through stress-induced hypercortisolemia, for instance, could result in downregulation of glucocorticoid receptors, which would, in turn, impair the negative feedback loop of cortisol. This would lead to dramatically enhanced levels of both corticotropin-releasing hormone (CRH) and adrenocorticotropic hormone (ACTH) [75]. Consistent dysregulation in this manner could lead to severe downstream complications, such as hippocampal atrophy, neurodegeneration, and reduced neurogenesis [73-75].

Metabolic disorders and depression

The association between obesity and depression may be in part due to shared pathophysiology and biological pathways.

A review of molecular and clinical gene-environment factors in the relationship between obesity and depression concluded that mediators of physiological stress may modulate this association [76]. Two prominent avenues of stress propagation include the interconnected norepinephrine system (a subsidiary of the sympathetic nervous system), and the HPA axis. The latter of these has been identified as a predominant biological pathway for the propagation of comorbid obesity and depression [76]. The HPA axis relies principally on the action of CRH, the locus coeruleus-norepinephrine autonomic systems, and the pituitary-adrenal axis [77]. Numerous studies have replicated this finding [74,75,78].

A 2011 review explored literature on the shared biological pathways between obesity and depression in an attempt to identify interconnected pathophysiology of the diseases [79]. Building upon the established evidence identifying both obesity and depression as chronic pro-inflammatory states, the authors focused particularly on aberrant inflammatory networks to elucidate the physiological basis of the bidirectional association [79]. They concluded that, based on their review, the association between these disorders may in fact be a manifestation of deviant inflammatory and/or neural networks.

Similarly, another research group also looked to interrelated immune system pathways in identifying the etiology of obesity and depression [80]. A review of literature on adipokines, cytokines secreted by adipose tissue, revealed multiple potential physiological pathways underlying the relationship between obesity and depression. Although research examining the biomarkers used by the authors remains scant, their results suggested a potentially significant role of adipokines in mediating the shared pathophysiology of the relationship between obesity and depression [80].

A more recent review of literature on this topic identified several overlapping pathways between obesity and depression that may underlie their shared pathophysiology [81]. Most prominent amongst these putative etiological hypotheses are aberrations in the HPA axis, dysfunctional brain-derived neurotrophic factor signaling, hormones and signaling molecules derived in adipose tissue, insulin signaling, the action of inflammatory cytokines, and oxidative and nitrosative stress pathways [81]. It is, of course, possible that the association between depression and obesity is propagated by numerous biological pathways, with varying levels of complexity and interrelatedness.

The association between obesity and depression may also be modulated by confounding variables, characteristic of either one of the diseases. For example, researchers have discovered that adolescents with a mutation in their cocaine and amphetamine-regulated transcript (CART) gene, which is significantly associated with early-onset obesity, may be substantially implicated in symptoms of anxiety and depression as well [82].

Genetics

In elucidating the association between these complex disorders, many researchers have turned to study genetic predispositions and factors.

Using a large, cross-sectional, multiethnic population from several distinct cohorts, one research group identified a protective effect of the obesity risk fat mass and obesity-associated (FTO) rs9939609 on depression [83]. This meta-analyzed inverse association (β 0.30 (0.08, 0.51); P = 0.0064) was adjusted for age, sex, ethnicity/population structure, and BMI. No significant between-study heterogeneity was identified (I2 = 0%; P = 0.63).

After establishing this paradoxically inverse association, the same research group attempted to investigate it with other obesity-related genetic variations [84]. The authors first established a positive association between BMI and depression status in a large, multi-ethnic, population cohort from the EpiDREAM study (OR = 1.02; 95% CI = 1.02-1.03 per BMI unit; P = 2.9 × 10-12). A total of 21 potential obesity-associated variants, other than FTO, were subsequently surveyed, and six of them were found to be significantly associated with BMI (1.47 × 10-14 ≤ P ≤ 0.04). Of these polymorphisms, TAL1 rs2984618 was associated with a higher risk of depression (P = 1.79 × 10-4), and brain-derived neurotrophic factor (BDNF) rs1401635 was significantly associated with depression in an ethnic-dependent manner (OR = 0.88; 95% CI, 0.80-0.97; P = 0.01 in non-Europeans and OR = 1.11; 95% CI, 1.02-1.20; P = 0.02 in Europeans; P-interaction = 2.73 × 10-4) [84]. The authors concluded that, based on their data, the observed relationship between obesity and depression could at least in part be explained by shared genetic factors.

A twin study of 712 monozygotic and 281 dizygotic female twin pairs explored a genetic link for the association between obesity and depression [85]. Obesity was defined as BMI ≥ 30, and depression status was self-reported based on prior physician diagnosis. A modest but statistically significant phenotypic association between the two conditions was established (OR = 1.6; 95% CI = 1.2-2.1). Both univariate and bivariate models were employed to estimate the components of variance attributable to genetic influences; the best fitting bivariate model identified that 12% of the genetic component of depression is shared with obesity [85]. The broader implications of this study are limited by the exclusivity of twin pair genders, as well as the significant role of shared environmental influences amongst twins. Nevertheless, the results provide a significant impetus for further study of genetic influences on the association between obesity and depression.

Building upon the known association between the FTO genotype and BMI, one group of researchers employed a Mendelian randomization approach to explore the association between longitudinal obesity and common mental disorders, including depression [86]. The study examined both men (N = 2,981) and women (N = 1,164), and only assessed the FTO rs1421085 polymorphism. Their results suggested that this FTO variant was associated with common mental disorders in men, but not in women. The established association was independent of adiposity.

Furthermore, in an attempt to elucidate a causal association between obesity and depression, one study examined the association between adult and adolescent BMI, and adult depressive symptoms [87]. A total of 31 single-nucleotide polymorphisms (SNPs), each of which was known to be a genetic marker of body weight, were used as proxies for variation in BMI. Linear regression analysis revealed that depressive symptoms as adults were significantly predicted by both adolescent (B = 0.33; 95% CI = 0.06-0.60; P = 0.017) and adult BMI (B = 0.47; CI = 0.32-0.63; P < 0.001). Instrumental variable analysis using genetic risk scores replicated these findings in both adolescents (B = 1.96; 95% CI = 0.03-3.90; P = 0.047) and adults (B = 1.08; 95% CI = 0.11-2.04; P = 0.030). These results suggest that obesity and increased BMI may indeed play a causal role in the onset of depression [87]. Similarly, another study also used instrumental variable analysis, using genetic variants as instrumental variables, to investigate the association between obesity and psychological distress [88]. Specifically, FTO rs9939609 and MC4R rs1778231 genetic loci were employed as instrumental variables for adiposity, as quantified by BMI and waist-to-hip ratio (WHR). Although multivariable analysis revealed a positive association between distress and both BMI and WHR, instrumental variables analyses suggested an inverse association between adiposity and psychological distress. This inverse relationship held true for both BMI (OR = 0.64, 95% CI = 0.46-0.89) and WHR (OR = 0.49; 95% CI = 0.25-0.94). These seemingly contradictory results may be explained by confounding and/or reverse causality in the conventional multivariable analysis. Regardless, this study further suggests the existence of potential biological and genetic pathways linking obesity and psychological distress [88].

To further investigate FTO and BMI within the context of depression, a 2012 study explored the influence of genetic variations in FTO on the major depressive disorder in two independent samples. A total of eight SNPs, each exhibiting a strong association with BMI, were investigated in population-based cohorts of depression cases and controls (2,442 major depressive disorder cases and 809 controls in the Radiant study, as well as 1,292 depression cases and 1,690 controls in the PsyCoLaus study). A significant interaction between seven of the eight SNPs and depression status in relation to BMI was determined in the Radiant study (P < 0.0057), and similar interaction for five of these SNPs was replicated in the PsyCoLaus cohort (P = 0.03-0.06). The meta-analyzed results indicate that the effect of FTO genotype on BMI is moderated by depression status. This in turn suggests that the association between obesity and depression is at least partially moderated by certain FTO variants [89].

Epigenetics

In addition to genetic, biological, and environmental sources of variability in the association between depression and metabolic disorders, another significant contributor includes epigenetic factors. Broadly, these include elements that modify DNA directly (for example, through DNA methylation), non-coding RNAs that modulate gene expression, and histone modifications (for example, through acetylation) [72,90].

Contemporary investigations have elucidated an extensive and complex role of epigenetics in the onset and proliferation of numerous psychiatric disorders [91,92]. Amongst these are depression and eating disorders, which include overeating and food addiction [93,94]. Such ailments, when exhibited in an obesogenic environment with access to excessive, palatable food options, can lead to obesity [93]. As depression co-occurs with eating disorders at a relatively high rate, it is possible that the latter at least partially confounds the association between depression and obesity. Therefore, epigenetic modifications that modulate the association can be present in known genetic and/or eating disorder genetic regions.

Two important epigenetic pathways implicated in increased susceptibility to obesity in later life have been identified. The mismatch pathway, involving an incongruency between early developmental environmental conditions and environmental conditions in later life, can result in an increased disposition to developing metabolic disorders. For example, mice that face severe environmental adversity in early life undergo epigenetic modifications to prepare for similar adversities to be faced in later life. For example, lack of sufficient nutrition during development can increase an organism’s sensitivity to obesogenic environments, particularly if environmental conditions in later life provide ample sources of nutrition [95]. Paradoxically, the second epigenomic pathway to late-life obesity involves maternal obesity and infant overfeeding. Evidently, the epigenomics of obesity are complex and not fully understood; nevertheless, great strides continue to be made in the field.

A systematic review of literature in 2013 revealed no substantial evidence for an association between global methylation of DNA and obesity [96]. The meta-analysis did, however, reveal differentially methylated sites associated with obesity, as well as a relationship between certain methylation marks at birth and obesity developed in later life [96].

Currently, epigenetic explanations for the association between obesity and depression have not been significantly explored. This domain remains, however, a salient area for future research.

## Conclusions

To summarize, we conducted a narrative review to investigate a link between depression and metabolic syndrome. With regards to obesity, it was shown to increase the risk of depression, while atypical depression increased the risk of obesity. Genetically, TAL1 rs2984618 and a mutation in the CART gene were found to be associated with both obesity and depression, along with the relationship being partially mediated by some FTO variants. Twin studies have also found that 12% of the genetic components of obesity and depression were shared amongst the two. Adipokines were also found to play a role in both obesity and depression. Disruption to the HPA axis was also found to be involved in both depression and obesity. The association between obesity and depression was also environmental as many factors like activity levels, self-esteem, and childhood adversities play a role. Epigenetically, while not extensively explored, depression and eating disorders share epigenetic changes, which, therefore, supports a link between obesity and depression.

With regards to diabetes, it was found to increase the risk of depression, while depressive symptoms increased the risk of type 2 diabetes. In addition, a number of environmental factors were found to be shared between diabetes and depression. Cardiovascular complications were also found to be linked to depression as physically healthy people had a 1.5-2 times higher risk of cardiovascular complications if suffering from depression.
